# Ameliorative effects of rutin against metabolic, biochemical and hormonal disturbances in polycystic ovary syndrome in rats

**DOI:** 10.1186/s13048-016-0295-y

**Published:** 2016-12-07

**Authors:** Sarwat Jahan, Faryal Munir, Suhail Razak, Anam Mehboob, Qurat Ul Ain, Hizb Ullah, Tayyaba Afsar, Ghazala Shaheen, Ali Almajwal

**Affiliations:** 1 Reproductive Physiology Laboratory, Department Of Animal Sciences, Quaid-i-Azam University, Islamabad, Pakistan; 2 Department of Community Health Sciences, College of Applied Medical Sciences, King Saud University, Riyadh, Saudi Arabia; 3 Department of Biochemistry, Quaid-i-Azam University, Islamabad, Pakistan

**Keywords:** Rutin, Letrozole, Polycystic ovary syndrome, Oxidative stress

## Abstract

**Background:**

Polycystic ovary syndrome (PCOS) is the most prevalent endocrinopathy in women of reproductive age. The study was commenced to assess the favorable effects of Rutin against metabolic, biochemical, histological, and androgenic aspects of polycystic ovary syndrome in rats.

**Methods:**

Female Sprague-Dawley rats were administered letrozole (1 mg/kg) per orally (p.o) for a period of 21 days for the induction of PCOS, followed by dose of rutin (100 mg/kg and 150 mg/kg, p.o) for 15 days using 0.5% w/v CMC as vehicle. Metformin was also given as a standard control to one of the rat groups.

Serum estradiol, progesterone, testosterone, serum lipid parameters, CRP and glucose levels were evaluated. Furthermore, antioxidant activity was tested using superoxide dismutase, catalase, glutathione per-oxidase and reactive-oxygen species level.

**Results:**

Rutin flavonoid had a dose-dependent effect on androgenic levels depicting more recovery in the rutin-I treated group, while rutin-II treated groups showed better antioxidant and lipid profiles as compared with PCOS groups. A decrease in the value of C reactive protein (CRP) and a restoration in the proportion of estrous phase smears were observed in the rutin treated groups. Histopathological examination of ovary revealed a significant decrease in the number of cystic follicles in post treated groups. The effects observed with rutin were moderately similar to that with standard metformin, a widely used treatment drug for PCOS.

**Conclusion:**

The study provides evidence for the potential ameliorative effects of rutin against clinical and biochemical features of PCOS.

## Background

Polycystic ovary syndrome (PCOS) is the most prevalent endocrinopathy in women of reproductive age. PCOS are distinguished by hyperandrogenism showing symptoms of hirsutism, acne, androgenic alopecia; irregularities of menstrual cycle such as polymenorrhea, oestrogen-replete amenorrhoea, metrorrhagia and oligomenorrhea, dyslipidemia, insulin resistance, chronic anovulation, hormonal imbalances and reduced fertility [1–4].

Various metabolic and clinical complications are associated with PCOS such as impaired glucose tolerance and diabetes, extensive coronary artery disease, hypertension, chronic oligo-ovulation, anovulation, infertility, endometrial, ovarian and breast cancers [5–11].

PCOS patients usually have a high oxidative profile which causes disturbance in antioxidants balance, leading to harmful effects of reactive oxygen species (ROS) including infertility, endometriosis, abortion, birth defects, preeclampsia, injury to ovarian epithelium’s DNA, excessive apoptosis and alteration in cell signalling process [12, 13].

Letrozole, known as Femara, inhibits the cytochrome P450 enzyme, aromatase, by the catalytic action of which estrogen is synthesized by the androgens. The absence of aromatase enzyme causes disturbance in the steroidogenesis, thus causing increase in the production of androgens developing PCOS [14].

Metformin is commonly known as Glucophage and it belongs to the biguanide class of drugs. It has the chemical formula of C_4_H_11_N_5_ and molecular weight of 129.1636 g/mol. Metformin suppresses the process of gluconeogenesis and results in the decrease in blood sugar levels, reduce body weight and may improve the *fibrinolysis* activity, lipid profile, insulin sensitivity and glucose uptake and utilization in peripheral tissues of skeletal muscle and adipocytes [15]. Metformin is also a best drug for PCOS treatment [16].

Flavonoids were chosen as a drug of choice for the cure of antiandrogenic, hyperlipidemic, hyperglycemic and oxidative stress consequences of PCOS. Flavonoids show a variety of biological actions including antimicrobial, antiviral, antiulcerogenic, cytotoxic, antineoplastic, anti-inflammatory, antioxidant, antihypertensive, hepatoprotective, hypolipidemic and antiplatelet activities [17].

The natural rutin (3′,4′,5,7-tetrahydroxyflavone-3-rutinoside) is one of the important phytochemicals. Rutin is a bioflavonoid which is necessary for the absorption of vitamin C and acts as an anti-oxidizer [18]. Rutin has a noteworthy range of scavenging characteristics on oxidizing species such as hydroxy radical, superoxide radical, and peroxyl radical by donating hydrogen atoms to peroxy radicals, superoxide anions, and singlet oxygen and hydroxyl radicals, it also functions as a terminator and chelator of metal ions that are able of oxidizing lipid peroxidation [19–21].

In this study, PCOS was induced by the use of aromatase inhibitor, letrozole, in the rodent model. Rutin was selected to be used as a therapy against polycystic ovary syndrome in the present study. Its comparative effects with metformin were assessed and it is anticipated to recover the complications caused due to PCOS.

The present study was designed to scrutinize whether rutin is effective in treating the endocrine, oxidative and reproductive dysfunctions in letrozole induced PCOS and to assess the prognostic power of this flavonoid in improving the clinical and biochemical features of PCOS.

## Methods

### Experimental animals

Six weeks old female Sprague-Dawley rats (*Rattus norvegicus*), having weight of about 155 ± 10 g and exhibiting normal estrous cycle of 6 days were taken from Animal house of Quaid-i-Azam University, Islamabad. Animals were supplied with pellets of feed and drinking water *ad libitum* and maintained in controlled experimental conditions in stainless steel cages. They were subjected to a 12:12 h light/dark cycles (relative humidity 60–65%) with room temperature of about 20 ± 5 °C for a period of 36 days. Animal handling, treatments and scarification was approved by the ethical committee of Department of Animal Sciences Quaid-i-Azam University, Islamabad.

PCOS was induced by oral administration of aromatase inhibitor, letrozole (1 mg/kg) dissolved in 0.5% CMC (2 mg/kg) for 21 days. This dose was selected using the previous studies of Kafali et al. [22] and Rezvanfar et al. [23]. During the experiment, the estrous cycle phases were monitored by the analyses of relative proportion of leukocytes, epithelial and cornified cells. All rats were randomly divided into following five groups consisting of five rats in each group: (i) control group (CMC; 2 mg/kg/day, p.o.), (ii) PCOS group (letrozole; 01 mg/kg/day, p.o.), (iii) Metformin group (metformin; 2 mg/100 g/day, p.o.), (iv) Rutin-I group (rutin; 100 mg/kg/day, p.o.), and (v) Rutin-II group (rutin; 150 mg/kg/day, p.o.). Letrozole and CMC were administered for 36 days while metformin and the two rutin doses were administered from day 21 to the day 36 of the experiment.

Rats were sacrificed on 37^th^ day, 24 h later the termination of the experiment. Blood was collected from the aorta of the rats under anesthesia in heparinized syringes and kept at -20 °C. It was then centrifuged at 3000 rpm for 15 min. Blood plasma was separated for biochemical and hormonal analysis and ovaries were taken out for histopathological and antioxidant assessments.

### Assessment of experimentally induced PCOS

#### Anthropometrical parameters

Changes in body weight were recorded every week in control, PCOS, metformin and rutin treated groups throughout the experiment.

Weight gain, body length, body mass index (BMI), the abdominal circumference to thoracic circumference (AC/TC) ratio, Lee index and specific rate of body mass gain were determined during the day of dissection using standard measurement methods.

Ovarian weight, diameter and ovarian organ index were also evaluated using usual measurement procedures. From the first day of study until termination, every morning colypocytological examination was performed to check estrous cyclicity in rats.

### Biochemical analysis

The concentration of blood glucose was evaluated on day 1, 21 and 36 of experiment using Accu Chek glucometer.

Antioxidant enzymes and protein levels were estimated in ovarian tissue of control and treated animals.

ROS value was determined using the protocol used by Hayashi et al. [24]. Catalase (CAT), Guaiacol peroxidase (POD) levels were analyzed as described in Chance and Maehly [25] with some modifications. Superoxide dismutase (SOD) was assessed by the protocol adopted by Kakkar et al. [26] and Thiobarbituric reactive acid substances (TBARS) levels were measured using Wright et al. [27] method. Gluthathione reductase (GSR); Carlberg & Mannervik [28], Glutathione-S-transferase (GST); Habig et al. [29], reduced Glutathione (GSH); Tietze et al. [30], Glutathione peroxidase (GSH-px); Mohandas et al. [31] and lipid hydroperoxide (LOOH) levels were measured using standard protocols used by (Jiang et al. [32]) on Smart Spec TM plus spectrophotometer.

Total cholesterol (TC), Triglycerides (TG), High-density lipoprotein (HDL-C), total protein and CRP were analyzed using commercially available kits of AMP diagnostics AMEDA Labordiagnostik GmbH, Austria. All the procedures were carried out using the manufacturer’s instructions.

Very low-density lipoprotein cholesterol (VLDL-C) and low density lipoprotein cholesterol (LDL-C) were calculated using Friedewald’s formula. Non-HDL cholesterol (non-HDL-C) was calculated as the difference between TC and HDL-C while TC/HDL, TG/HDL and LDL/HDL were also assessed.

### Hormonal analysis

Serum total testosterone, progesterone and estradiol were evaluated using commercially available ELISA kits of Microlisa AMGENIX Int, Inc. USA. All the procedures were carried out using manual instructions.

### Histopathological analysis

The ovaries were processed for paraffin embedding, sectioned at 7 μm, stained with hematoxylin and eosin, and observed under microscope at 100 × s magnification. All the ovarian follicles were examined depending upon their granulosa and morphology. The presence or absence of corpus luteum and thickness of peripheral theca and granulosa layer were also observed.

### Statistical analysis

Data were represented as Mean ± Standard Error of Mean (SEM). Graph Pad PRISM 5 (San Diego, CA, USA) was used to describe the results statistically. Comparison of mean was done by using one-way analysis of variance (ANOVA), followed by Tukey test.

## Results

### Effect of metformin and rutin on anthropometrical parameters

Administartion of letrozole to female rats for 36 days resulted in a significant increase in final body weights of PCOS group as compared to control (*P* < 0.05), however all the other anthropometrical parameters including weight gain, AC, TC, AC/TC ratio, BMI, Lee index and specific rate of body mass gain remained non-significant among rats in the various groups. Body length showed a significant increase in the PCOS group as compared to control group (*P* < 0.05) (Table 1).

**Table 1:** Effect of Rutin-I (100 mg/kg) and Rutin-II (150 mg/kg) on anthropometrical parameters in Letrozole induced PCOS rats after 36 days of experiment

Parameters	Control	PCOS	Metformin	Rutin-I	Rutin-II
Weight gain (g)	25.20 ± 3.32	62.00 ± 10.61	46.20 ± 8.73	52.40 ± 5.16	36.01 ± 15.72
AC	14.78 ± 0.42	15.82 ± 0.27	15.74 ± 0.29	15.2 ± 0.34	15.02 ± 0.29
TC	13.86 ± 0.27	14.94 ± 0.18	14.78 ± 0.29	14.92 ± 0.18	14.36 ± 0.15
AC/TC ratio	1.06 ± 0.02	1.05 ± 0.01	1.06 ± 0.01	1.01 ± 0.01	1.04 ± 0.02
Body Length (cm)	19.24 ± 0.35	20.62 ± 0.36^a*^	20.42 ± 0.29	20.50 ± 0.27	20.12 ± 0.28
Body mass Index (g/cm^2)^	0.491 ± 0.016	0.545 ± 0.023	0.477 ± 0.018	0.522 ± 0.025	0.493 ± 0.029
Lee Index	0.294 ± 0.004	0.292 ± 0.006	0.288 ± 0.003	0.293 ± 0.005	0.287 ± 0.004
Specific Rate of Body mass gain (g/kg)	4.343 ± 0.945	27.760 ± 8.616	16.211 ± 5.041	26.660 ± 5.475	16.170 ± 9.213

AC/TC, abdominal circumference to thoracic circumference; PCOS, polycystic ovary sundrome

Values are expressed as Mean ± SEM

^*^ *p*< 0.05

^a^Value vs control

### Effect of metformin and rutin on colpocytological examination

Administration of letrozole to rats for 36 days caused disturbance of normal estrous cycle starting from day 17^th^ till the end, in the PCOS group, resulting into a profuse vaginal secretion. Mainly diestrus stage remained dominant in it, exhibiting irregularity of the estrous cycle as compared to control group. In rats treated with metformin, rutin (100 mg/kg) and rutin (150 mg/kg), the normal estrous cycle depicted a restoration of regular phases when compared with control and PCOS groups.

### Effect of metformin and rutin on weights of ovaries

There was no significant difference noted in weights, diameter and ovarian organ index among the different groups (Table 2).

**Table 2:** Effect of Rutin-I (100 mg/kg) and Rutin-II (150 mg/kg) on weights, diameter and ovarian organ index of ovaries in Letrozole induced PCOS rats after 36 days of experiment

Groups (*n*=5)	Control	PCOS	Metformin	Rutin-I	Rutin-II
Weight of left ovary (mg)	57.03 ± 3.23	66.67 ± 4.53	57.67 ± 5.08	66.33 ± 2.54	61.44 ± 4.23
Weight of right ovary (mg)	56.01 ± 2.44	65.25 ± 5.09	59.20 ± 4.01	64.03 ± 6.78	60.41 ± 3.72
Diameter of right ovary (mm)	4.80 ± 0.37	5.10 ± 0.24	4.60 ± 0.24	5.00 ± 0.31	4.80 ± 0.20
Diameter of left ovary (mm)	5.00 ± 0.44	5.60 ± 0.24	5.20 ± 0.20	5.50 ± 0.22	5.30 ± 0.20
Ovary organ index	0.31 ± 0.01	0.29 ± 0.02	0.28 ± 0.01	0.29 ± 0.01	0.31 ± 0.02

Values are expressed as Mean±SEM

### Effect of metformin and rutin on glucose concentration

On the initial day of experiment blood glucose levels were measured. Mean ± SEM is given in Table 3. There was no major change in the mean values among the groups on the initiation of PCOS induction. On day 21, before the start of post treatment, glucose levels were again measured. PCOS and metformin group depicted significant increase (*P* < 0.001) when compared with control group, whereas, rutin–I and rutin–II groups illustrated significant increase (*P* < 0.05) as compared to control group. On the termination of experiment i.e., on day 36, highly significant increase (*P* < 0.001) in glucose concentration was noticed in PCOS group as compared to control group. On contrary to this, the metformin, rutin-I (100 mg/kg) and rutin-II (150 mg/kg) post treated groups showed highly significant decrease (*P* < 0.001) when compared with PCOS group.

**Table 3:** Effect of Rutin-I (100 mg/kg) and Rutin-II (150 mg/kg) on glucose levels measured on day 1, 21 and 36 of experiment in Letrozole induced PCOS rats

Glucose levels (mg/dL)	Control	PCOS	Metformin	Rutin-I	Rutin-II
Day 1	74.21 ± 1.93	73.23 ± 2.81	73.40 ± 3.29	73.01 ± 2.98	72.61 ± 2.42
Day 21	79.22 ± 3.21	95.61 ± 3.18^a**^	95.83 ± 1.93^a**^	93.21 ± 3.30^a*^	92.80 ± 2.72^a*^
Day 36	79.20 ± 3.11	96.44 ± 2.00^a***^	77.21 ± 1.90^b***^	79.80 ± 1.01^b***^	76.40 ± 1.60^b***^

Values are expressed as Mean ± SEM

*=*p*< 0.05, **=*p*< 0.01, ***=*p*< 0.001

^a^Value vs control, ^b^Value vs PCOS

### Effect of metformin and rutin on total protein and C reactive protein

In PCOS group, the total protein values increased significantly as compared with the control group (*P* < 0.05) while they were significantly reduced in the rutin-II group (*P* < 0.05) when compared with PCOS group.

The values of C-reactive protein (CRP) increased significantly (*P* < 0.05) in the PCOS group when compared with the control group. None of the other groups i.e., metformin, rutin-I (100 mg/kg) and rutin-II (150 mg/kg) exhibited any significant change in the CRP values when compared with control, PCOS and among themselves (Table 4).

**Table 4:** Effect of Rutin-I (100 mg/kg) and Rutin-II (150 mg/kg) on total protein and CRP concentration in Letrozole induced PCOS rats after 36 days of experiment

Protein values	Control	PCOS	Metformin	Rutin-I	Rutin-II
Total Protein (mg/g of tissue)	25.371 ± 1.852	40.242 ± 1.771^a*^	25.491 ± 2.423^b*^	26.701 ± 6.264	25.342 ± 2.944^b*^
CRP (mg/dL)	0.051 ± 0.004	0.069 ± 0.001^a*^	0.060 ± 0.006	0.062 ± 0.002	0.059 ± 0.004

Values are expressed as Mean ± SEM

*p< 0.05

^a^Value vs control

^b^Value vs PCOs

### Effect of metformin and rutin on ROS

The value of ROS in PCOS group increased significantly as compared to that of control group (*P* < 0.05) but the change was not significant when taken into comparison with metformin and rutin groups. The values of metformin, rutin-I and rutin-II groups varied significantly as compared with that of the PCOS group (*P* < 0.05). But there was non-significant change in metformin, rutin-I and rutin-II groups as compared with that of the control group and also among each other (Table 5).

**Table 5:** Effect of Rutin-I (100 mg/kg) and Rutin-II (150 mg/kg) on ROS, TBARS, LOOH, CAT, POD, SOD, GSR, GST, GSH, GSH-px concentrations in Letrozole induced PCOS rats after 36 days of experiment

Parameters	Control	PCOS	Metformin	Rutin-I	Rutin-II
ROS (U/min)	0.20 ± 0.04	0.39 ± 0.09^a*^	0.17 ± 0.02^b*^	0.19 ± 0.02^b*^	0.16 ± 0.01^b*^
TBARS (nmol/min/mg)	5.69 ± 1.07	16.06 ± 1.25^a**^	10.78 ± 1.07	8.93 ± 2.45^b*^	8.15 ± 0.82^b*^
LOOH (nmol/min/mg)	3.29 ± 0.48	5.39 ± 0.29^a*^	3.44 ± 0.47	3.59 ± 0.63	3.23 ± 0.42^b*^
CAT(U/min)	4.93 ± 0.08	3.49 ± 0.33^a*^	4.15 ± 0.47	4.22 ± 0.28	4.49 ± 0.38
POD(nmole)	12.09 ± 0.72	9.29 ± 0.01^a*^	10.44 ± 0.62	10.11 ± 0.58	11.87 ± 0.59^b*^
SOD (U/min)	5.81 ± 0.24	2.74 ± 0.29^a*^	5.51 ± 0.51^b*^	5.41 ± 1.08^b*^	5.72 ± 0.51^b*^
GSR (U/mol/mg)	3.96 ± 0.38	1.79 ± 0.09^a*^	3.89 ± 0.29^b*^	3.46 ± 0.71	4.67 ± 0.52^b*^
GST (U/mol/mg)	10.85 ± 0.21	8.02 ± 0.83^a**^	9.47 ± 0.28	9.37 ± 0.48	10.91 ± 0.22^b*^
GSH (U/mol/mg)	6.91 ± 0.50	4.97 ± 0.14^a**^	6.55 ± 0.48^b*^	6.39 ± 0.02^b*^	6.77 ± 0.88^b**^
GSH-px (U/mol/mg)	29.87 ± 2.14	20.35 ± 0.64^a**^	25.34 ± 2.11	24.24 ± 1.76	30.10 ± 1.29^b**^

Values are expressed as Mean ± SEM

*=*p*< 0.05, **=*p*< 0.01

^a^Value vs control, ^b^Value vs PCOS

### Effect of metformin and rutin on antioxidant profile

The results regarding the protective effects of rutin against PCOS in rat ovaries and activities of antioxidant enzymes such as CAT, POD, SOD, TBARS, LOOH, GSH-Px, GSR, GSH and GST are shown in Table 5. Activities of antioxidant enzymes such as CAT, POD, SOD, GSR, GST, GSH and GSH-Px were significantly reduced in PCOS group as compared to control group. This reduction in enzymes activity was reversed non significantly by the treatment of metformin while significant increase was seen in the values of SOD, GSR and GSH (*P* < 0.05). Rutin (100 mg/kg) did not show any significant difference (*P* < 0.05) among various groups except the significant increase in the SOD and GSH value as compared to the PCOS group. Alteration in the ovarian enzyme activities with the treatment of Rutin (150 mg/kg) showed statistically significant increase (*P* < 0.05) as compared to PCOS group.

Letrozole increased the TBARS (*P* < 0.01) and LOOH (*P* < 0.05) levels significantly in PCOS as compared to control. Metformin did not show any significance while rutin (100 mg/kg), rutin (150 mg/kg) treatment exhibited significant decrease (*P* < 0.05) in both antioxidants.

### Effect of metformin and rutin on serum testosterone, progesterone and estradiol values

Administration of letrozole to female rats for 36 days resulted in a highly significant increase in the serum testosterone as compared with the control (*P* < 0.001) while the metformin, rutin-I (100 mg/kg) and rutin-II (150 mg/kg) exhibited a moderately significant decrease (*P* < 0.01) in comparison with the PCOS group but remained non-significant when compared with the control group. There was no significant difference in the values of estradiol among rats of all the groups. Letrozole administration resulted in a very significant decrease (*P* < 0.01) in the progesterone value in the PCOS group when compared with the control group (Table 6).

**Table 6:** Effect of Rutin-I (100 mg/kg) and Rutin-II (150 mg/kg) on serum testosterone, estradioland progesterone concentrations in Letrozole induced PCOS rats after 36 days of experiment

Hormonal concentrations	Control	PCOS	Metformin	Rutin-I	Rutin-II
Testosterone (ng/ml)	1.88 ± 0.71	10.19 ± 0.76^a***^	4.51 ± 0.30 ^b**^	5.77 ± 0.68^b*^	8.21 ± 1.85^a**^
Estradiol (ng/ml)	19.41 ± 2.21	14.26 ± 2.70	28.57 ± 4.32	34.83 ± 8.26	27.25 ± 0.48
Progesterone (pg/ml)	43.43 ± 8.81	12.76 ± 3.75^a**^	22.98 ± 4.85	19.66 ± 5.34^a*^	24.63 ± 3.46

Values are expressed as Mean ± SEM

*=*p*< 0.05, **=*p*< 0.01, ***=*p*< 0.001

^a^Value vs control, ^b^Value vs PCOS

### Effect of metformin and rutin on lipid profile

Administration of letrozole (1 mg/kg) to female rats for 36 days resulted in a significant increase in serum TG, VLDL-C, LDL-C and non-HDL-C, whereas HDL-C was significantly decreased when compared with vehicle control (*P* < 0.05). Metformin significantly reduced TC (*P* < 0.05), LDL-C and non-HDL-C (*P* < 0.01). Rutin-I (100 mg/kg) treatment caused significant reduction in TC and non-HDL (*P* < 0.05). While rutin-II (150 mg/kg) significantly lowered TC, TG, VLDL-C, LDL-C and non-HDL-C but did not affect the level of HDL-C (Table 7).

**Table 7:** Effect of Rutin-I (100 mg/kg) and Rutin-II (150 mg/kg) on total cholesterol, triglycerides, HDL- C, LDL-C, VLDL-C, Non-HDL-C, TC/HDL, TG/HDL and LDL/HDL concentrations in Letrozole induced PCOS rats after 36 days of experiment

Parameters	Control	PCOS	Metformin	Rutin-I	Rutin-II
Total Cholesterol(mg/dL)	215.60 ± 3.43	231.10 ± 4.27	211.30 ± 6.81^b*^	212.21 ± 1.53^b*^	207.10 ± 2.24^b**^
Triglycerides (mg/dL)	185.11 ± 4.23	213.10 ± 5.16^a*^	197.40 ± 5.82	199.20 ± 6.93	186.12 ± 4.62^b*^
HDL-C (mg/dL)	160.70 ± 4.17	144.41 ± 3.63^a*^	153.21 ± 1.80	146.43 ± 4.52	159.80 ± 3.14^b*^
LDL-C (mg/dL)	17.83 ± 3.98	42.99 ± 1.99^a**^	18.58 ± 7.22^b**^	25.81 ± 5.11	10.05 ± 2.29^b***^
VLDL-C (mg/dL)	37.03 ± 0.85	42.61 ± 1.03^a*^	39.49 ± 1.16	39.83 ± 1.39	37.20 ± 0.92^b*^
Non-HDL-C(mg/dL)	54.86 ± 4.48	85.61 ± 1.50^a**^	58.07 ± 7.32^b**^	65.65 ± 4.75^b*^	47.24 ± 2.64^b***^
TC/HDL	1.34 ± 0.03	1.59 ± 0.02^a***^	1.38 ± 0.05^b**^	1.45 ± 0.04	1.29 ± 0.02^b***c*^
TG/HDL	1.16 ± 0.04	1.48 ± 0.06^a**^	1.29 ± 0.03	1.37 ± 0.06	1.17 ± 0.04^b**^
LDL/HDL	0.11 ± 0.03	0.29 ± 0.01^a**^	0.12 ± 0.05^b**^	0.18 ± 0.04	0.06 ± 0.02^b***^

Values are expressed as Mean ± SEM

* = *p* < 0.05, ** = *p* < 0.01, *** = *p* < 0.001

^a^ Value vs control, ^b^ Value vs PCOS, ^c^ Value vs Metformin

### Effect of metformin and rutin on histopathology of ovary

**Fig. 1:**
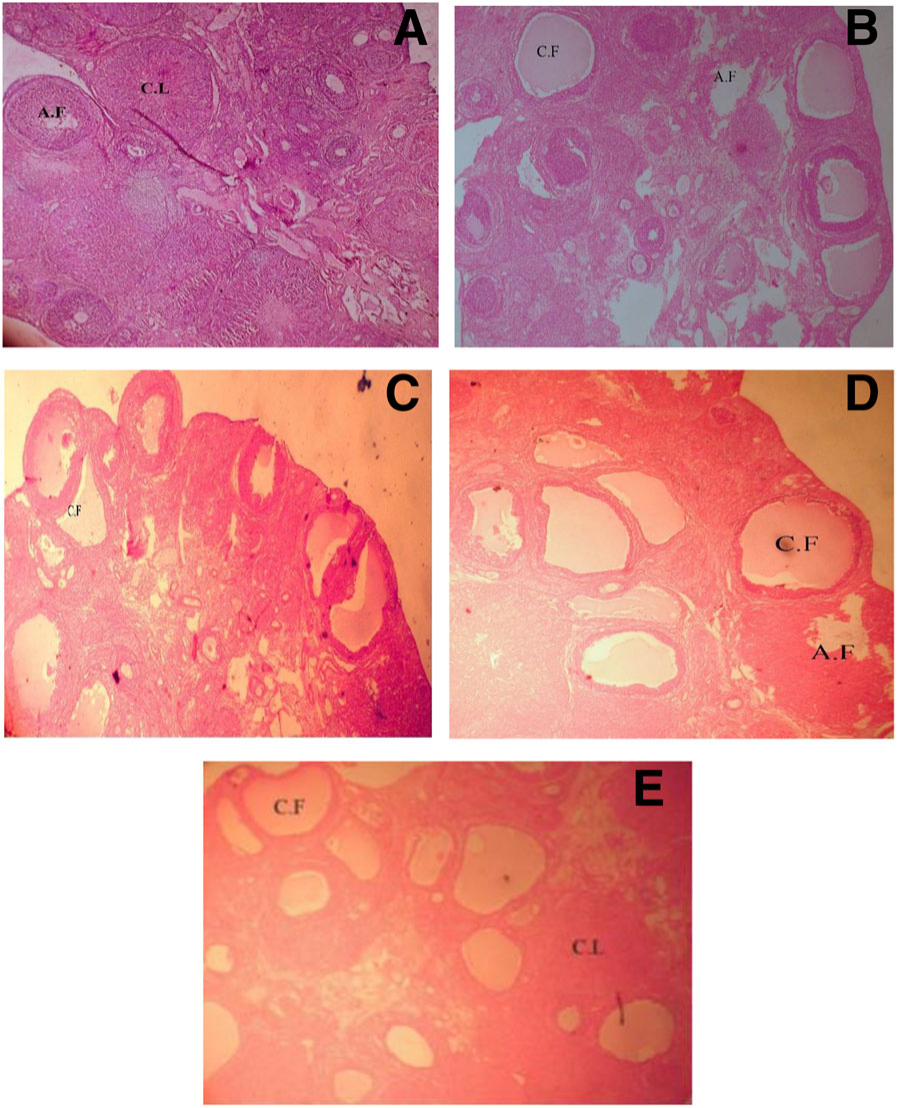
Histopathological features of ovary in Letrozole induced PCOS in rats. Representative photographs of section of ovary showing various developing follicles, cystic follicle (c.f), atretic follicle (a.f) and corpus luteum (c.l): (**a**) Control group (**b**) PCOS group (**c**) Metformin group (**d**) Rutin-I (100 mg/kg) and (**e**) Rutin-II (150 mg/kg) (40× magnification)

The histological changes studied were as follows: (Fig. 1).

### The diameter of developing follicles

The diameter of developing follicles (D20 μm –D > 600 μm) did not depict any significant difference among the various groups.

### Number of cystic and atretic follicles

The number of cystic and atretic follicles were increased significantly (*P* < 0.001) in the PCOS group as compared with the control group. Metformin, rutin (100 mg/kg) and rutin (150 mg/kg) significantly reduced the number of cystic follicles (*P* < 0.001) and atretic follicles (*P* < 0.05) as compared to PCOS group (Table 8).

**Table 8:** Effect of Rutin-I (100 mg/kg) and Rutin-II (150 mg/kg) on number of cystic follicles, atretic follicles and corpus luteum in Letrozole induced PCOS rats after 36 days of experiment

Number of follicles	Control	PCOS	Metformin	Rutin-I	Rutin-II
Cystic follicles	0.0 ± 0.0	10.17 ± 0.70^a***^	5.16 ± 0.30^a***b***^	6.16 ± 0.47 ^a***b***^	7.33 ± 0.42^a***b**c*^
Atreticfollicles	2.00 ± 0.25	4.50 ± 0.34^a***^	2.83 ± 0.47^b*^	2.66 ± 0.33^b*^	2.83 ± 0.40^b*^
Corpousluteum	6.80 ± 0.37	2.40 ± 0.24^a***^	4.20 ± 0.58^a**b*^	2.40 ± 0.50^a***^ ^c*^	4.40 ± 0.24^a**b*d*^

Values are expressed as Mean ± SEM

*p< 0.05, **p< 0.01, ***p< 0.001

^a^Value vs control, ^b^Value vs PCOS, ^c^Value vs Metformin, ^d^Value vsRutin-I

### Number of corpus luteum

The number of corpus luteum was significantly decreased in the PCOS group as compared to control (*P* < 0.001) whereas significant recovery was shown in the metformin, rutin-I and rutin-II groups (*P* < 0.05) (Table 8).

### Thickness of peripheral thecal and granulosa layer in normal and cystic follicles

The thickness of peripheral thecal layer (D200 μm -D > 600 μm) remained non-significant in the normal follicles among the various groups but its thickness increased significantly (*P* < 0.001) in the PCOS group when compared with control group. The thickness was restored significantly (*P* < 0.001) in metformin and rutin post treated groups when compared with PCOS group.

PCOS group showed highly significant (*P* < 0.001) reduction in peripheral granulosa thickness in follicles when compared with control group whereas in cystic follicles width of granulose layer increased non-significantly in metformin, rutin-I and rutin-II group as compare to PCOS group (Table 9).

**Table 9:** Effect of Rutin-I (100 mg/kg) and Rutin-II (150 mg/kg) on thickness of thecal layer and peripheral granulosa layer in Letrozole induced PCOS rats after 36 days of experiment

Thickness (μm) Rutin I	Control Rutin II	PCOS	Metformin
Thecal Layer	–	27.56±2.10	21.25±3.09
18.11±1.28^a***^	16.78±1.25^a^***		
Granulosa Layer	–	22.00±2.55	30.00±2.73
26.00±2.91	25.00±3.16		

Values are expressed as Mean ± SEM

***=*p*< 0.001

^a^Value vs PCOS

## Discussion

Letrozole, an aromatase inhibitor, was used to induce PCOS in the rats. Subsequently the increased weights of rats and irregular estrous cycle in the positive control substantiated the induction of PCOS and also signified that the rat model is anoestrous, as it imitated anovulatory characteristic [33]. Treatment of PCOS induced rats with flavonoid rutin possibly restored the estrous cyclicity in rats. The rutin treated groups, however, did not depict any significant increase in body weight which suggested that rutin might have reduced the expression levels of adipogenic genes thus reducing the weight of adipose tissues [34].

Surprisingly, the ovarian weights, ovarian diameter and ovarian organ index displayed no significant increase. The non-significant anthropometrical results might be due to the fast metabolic response of the animals, suggesting that other genetic or environmental factors or longer time of exposure to aromatase inhibitor was necessary to achieve the full spectrum of ovarian pathology.

The glucose levels increased significantly when measured in the PCOS group during the day 21 and 36 of the experiment. Letrozole seems to create a disturbance in the hormonal profile of the animals mainly because of increased androgen levels. This increased androgen levels in turn induced insulin resistance hence creating a decreased glucose tolerance [35]. Meanwhile, metformin reduced the glucose levels significantly as compared to PCOS group. This may be due to the fact that metformin treatment decreased the glucose resistance by maintaining the glucose homeostasis and also by improving the insulin-mediated uptake of glucose [35, 36]. Rutin flavonoid significantly reduces the glucose values in the treated groups as compared with PCOS group because of its antihyperglycemic effect and caused a decrease in the glycaemic levels by conceivably playing its role as potentiating the insulin secretion by the β-cells of Islets of Langerhans thus promoting a balanced uptake of glucose by the cells [37]. It also suggested that rutin might be a potential agent for glycaemic control through enhancement of insulin-dependent receptor kinase activity, thus inducing the insulin signalling pathway, and consequently causing increased glucose transporter 4 translocation and increased glucose uptake [38].

The total protein index increased significantly in the PCOS group. This abnormal level of total protein might suggest infectious and chronic inflammation in the PCOS group which might be due to the abnormal androgen secretions. However, metformin significantly lowered the amount of total protein as compared with the positive vehicle group. The improved sensitivity of proteins and protein hormones after the metformin therapy down regulate the increased circulating levels of protein in the blood [39]. Rutin flavonoid significantly decreased the total protein level by inhibiting the eicosanoids and cytokines due to its pharmacologic actions [40]. The C- reactive protein (CRP) is an acute phase protein and a very expedient indicator of inflammation in a tissue. In present study, the CRP levels increased significantly in the PCOS group thus exhibiting severe infection [41].

Elevated testosterone levels in PCOS most probably reflect build-up of androgens because conversion of androgen substrates into estrogens was blocked by aromatase inhibitor. The decrease in testosterone concentration in metformin group reflect diminished androgen biosynthesis by the ovary [42]. The diminution of estrogen production by aromatase inhibition can cause enhanced secretion of LH in hypothalamus and pituitary most likely by negative feedback of estrogens.

Oxidative stress is generally regarded as the phenomena where the generation of reactive oxygen species (ROS) cause disruption in the normal functionality of biological system. The aerobic cells have a counter mechanism to deal with oxidative stress by trapping the reactive oxygen intermediates but the inequity in terms of the rate of the production of ROS and their protective systems can cause oxidative stress over time. In present study, the ROS level increased significantly in the PCOS group which depicted an increase in the oxidative stress [43]. The increase in ROS level indicates the molecular damage in the cellular structure and the tissue was mainly due to the over production of free oxidative radicals. ROS value was also decreased significantly in the rutin groups, which is due to the antioxidant activity of rutin. The exact mechanism through which rutin induces antioxidative action is not clear but it reduces the causative agents of oxidative stress [44].

Lipid peroxidation is a free-radical mediated promulgation of oxidative offence inserted to polyunsaturated fatty acids involving numerous types of free radicals. The cessation of lipid peroxidation occurs by antioxidants through their enzymatic action or by free radical scavenging activity [45]. The increase in the lipid per hydroxide levels might be due to the termination of antioxidant effect which caused pathogenesis of the disease.

Histopathological observation of ovaries taken from Letrozole treated animals displayed remarkable resemblance to human PCOS, revealing sub capsular cysts lined with a thin layer of granulosa cells and hyperplasia of theca cells [12]. It has been observed that the increased levels of intra-ovarian androgens lead to abnormal follicular growth and an increase in follicular artesia [46, 47]. Number of corpus luteum and different developing follicles measuring in diameter from 20 μm to more than 600 μm also decreased non-significantly in PCOS group. Cystic follicles were much larger in size as compared to other follicles of ovary with a clear antrum but lacked oocyte. Thickness of peripheral granulosa layer was decreased while that of theca layer was increased significantly in different follicles of PCOS group, as compared to control group. Contrary to this, post treated groups exhibited non-significant increase when compared with PCOS group. These histological findings were pinpointing towards the existence of biologically active levels of FSH, increased LH, and lack of interplay between granulosa and theca cells, which would otherwise lead to ovulation, in the PCOS group.

## Conclusions

Concluding our work, present study demonstrates antiandrogenic properties of rutin. It showed estrogenic, antiandrogenic and anti hyperglycemic effects and recovered ovarian cysts and its protective properties was seen to be comparable to that of metformin. The restoration of ovarian function and hormonal profile by rutin treatment could be supportive in managing PCOS. Outcomes of the study also revealed that letrozole induced PCOS had an enhanced level of oxidative stress along with hyperglycemia and hyperlipidemia. Oxidative stress in turn damaged the tissue by reactive oxygen species (ROS) and the rutin flavonoid was able to antidote the oxidative stress. Meanwhile, rutin also played an imperative role in reducing the oxidative stress, boosting the antioxidant status, and possibly reducing the ROS and lipid peroxidation. It displayed a significant role in subsiding the hyperlipidemic state and hyperglycemic state and it can be used as a possible ameliorative medication for curing clinical and biochemical characteristics of polycystic ovary syndrome, its properties were analogous to that of standard metformin. Further research is prerequisite to investigate the pharmacologic and therapeutic potentials of rutin so that it can be used as an adjunct therapy sideways to currently used drugs in PCOS management.
